# Characterizing the Rheological and Bread-Making Properties of Wheat Flour Treated by “Gluten Friendly^TM^” Technology

**DOI:** 10.3390/foods10040751

**Published:** 2021-04-01

**Authors:** Carmela Lamacchia, Loretta Landriscina, Carla Severini, Rossella Caporizzi, Antonio Derossi

**Affiliations:** Department of Agriculture, Food, Natural resources and Engineering (DAFNE), University of Foggia, 71122 Foggia, Italy; loretta.landriscina@unifg.it (L.L.); carla.severini@unifg.it (C.S.); rossella.caporizzi@unifg.it (R.C.)

**Keywords:** Gluten Friendly^TM^ technology, microwaves, gluten friendly bread, rheological properties, pasting properties, sensorial evaluation

## Abstract

After discovering an innovative technology for the reshaping of gluten proteins—the “Gluten Friendly^TM^” system—that confers to wheat flour some unprecedented characteristics, such as reduced epitope antigenicity and a positive modulation of the gut microbiota, its effects on the production and quality of bread have been studied. Mainly, we have investigated the chemical, rheological and pasting properties of Gluten Friendly Flour (GFF) and of control flour (CF) with the aim of analyzing and interpreting potential differences. Furthermore, the bread made from GFF and CF was evaluated in terms of microstructure properties and sensory quality. The experiments demonstrated that GFF became soluble in aqueous solution, making it unfeasible to isolate using the Glutomatic apparatus. Although the water absorption of GFF increased by 10% compared to CF, dough elasticity was reduced, and dough stability decreased from 5 to 2 min. A significant increase in the alveograph index (P/L) from 0.63 to 6.31 was detected, whereas pasting properties did not change from the control flour. Despite these profound modifications in the rheological properties, GFF exhibited a high ability to shape dough and to produce bread with high quality and negligible differences from the control bread in terms of appearance, taste, aroma, color and texture.

## 1. Introduction

Coeliac disease (CD) is an autoimmune disease triggered by the cereal protein gluten [1,2]. The most recent medical data indicates that approximately 1% of the global population suffers from CD, corresponding to 69 million people [3]. Considering European citizens, only 25% of people suffering CD have received a correct diagnosis, leading to a defined prevalence of 1:100, whereas in 2003 the recognized prevalence was 1:200 [2,4].

For these subjects, the consumption of cereals containing gluten, such as wheat, barley and rye, causes a chronic inflammatory process, generating severe lesions on the small intestine, dysfunctions in nutrient absorption and a series of non-gastrointestinal symptoms that withdraw when the gluten exposure is interrupted [1,5]. For these reasons, the only solution capable of fully avoiding chronic disease is the adoption of a strictly gluten-free diet throughout the patient’s lifetime. Although this dietary regimen guarantees the full recovery of small intestine architecture and functions, this solution may be difficult to follow as it is strongly restrictive, especially when people are eating out or during social events and travelling, where the risk of cross-contamination may also significantly increase [6].

In recent years, the food industry has successfully satisfied the increasing demand for gluten-free products, classified as products with a gluten concentration <20 mg/Kg [7] and has made them recognizable on the market with the Crossed Grain symbol. To date, the Association of European Coeliac Societies have licensed more than 22,000 gluten free products for coeliac patients [4], demonstrating the high interest of the food companies in manufacturing nutritious and tasty gluten-free foods. However, some concerns remain as to the daily employment of gluten-free food products [8]. Considering their nutritional properties, because gluten-free products are generally produced with rice and maize and due to malabsorption caused by intestinal damage, coeliac patients are widely affected by some nutritional deficiencies, among which calcium, iron, copper, vitamin B, magnesium and zinc deficiencies are the most noticeable [9,10]. For this reason, the use of unusual gluten-free grains like amaranth, teff, quinoa and buckwheat is steadily increasing and several detailed experiments have been published [8,11,12,13]. However, when gluten-free grains are utilized, significant behavioral changes are observed during food processing, mainly in their technological performance (i.e., viscosity and elasticity), which leads to significant diversities in their structural, textural and sensorial attributes, as recognized worldwide [14,15,16]. For instance, the lack of gluten reduces the amount of gas bubbles entrapped in the dough matrix and hinders the desired increase in volume during leavening and baking [17]. All this reduces the flavor and the palatability of cereal-based products obtained by gluten-free grains. To tackle this challenge, several technological strategies have been studied to fully prevent or to reduce gluten toxicity without any loss of technological and sensory performance. The majority of the proposed solutions involve the use of peptidase, which leads to a complete degradation of toxic peptides [18,19] or the transamidation of toxic epitopes [20,21,22].

Recently, a new innovative detoxification method for gluten proteins in cereal grains (Italian patented method n°: 0001414717, also filed under the Patent Cooperation Treaty, application no. PCT/IB2013/000797) has been developed by Lamacchia, Di Luccia and Gianfrani [23,24]. This innovation is popularly referred to as “Gluten Friendly™” technology, and by applying microwave energy for a few seconds to the hydrated wheat kernels, it induces structural changes in proteins capable of eradicating the antigenic capacity of gluten [25,26] and to reduce the immunogenicity in vitro of the most common epitopes involved in coeliac disease [27]. Furthermore, many healthy effects have been observed, such as the enhancement of the qualitative and quantitative composition of the microbiota of coeliac people in a model system [28], better modulation of the fecal microbiota and short chain fatty acids [29], as well as the improvement of intestinal epithelial barrier function and mucin secretion in an in vitro experiment [6].

However, despite the great positive impact of “Gluten Friendly™” technology on healthy properties, experiments focused on its effects on technological performance, such as rheology of doughs and bread-making characteristics, are still limited [25].

The aim of this paper is to address this gap by performing detailed experiments on the effects of “Gluten Friendly™” technology on the most important bread-making properties. To do this, the rheological behavior of doughs prepared by “Gluten Friendly™” wheat flours in comparison with common untreated wheat flours were analyzed. Furthermore, innovative and traditional bread samples were investigated to describe their microstructure characteristics and to define their levels of sensory acceptance.

## 2. Materials and Methods

### 2.1. Raw Materials and Microwave Treatment

Wheat kernels (mixtures of Canadian grains) used in this study to prepare “Gluten Friendly™” (GF) flour were supplied by Casillo group S.p.A. (Corato, Italy). The grains were harvested and threshed, then treated with microwave energy according to the patented “Gluten Friendly™” technology [23]. The technology has since been further improved according to Italian priority patent n° 102015000084813 [30]. Specifically, 100 g of cleaned wheat grains were dampened to achieve 15–18% moisture; moisture was evaluated using a Halogen Moisture Analyzer (Mettler Toledo HB43-S, Greifensee, Switzerland). The kernels were then heated with microwaves (DeLonghi, Treviso, Italy, for about 1 min between 1000 and 750 W), followed by a phase of slow evaporation of water content. Rapid heating and slow evaporation were repeated until reaching a temperature of 80–90 °C, as measured with a thermal camera (FLUKE i 20, Brughiero, Italy), and a moisture level of 13–13.5%. After microwave treatment, GF wheat kernels were cooled and dried at room temperature (24 °C) for 12–24 h, and then ground with an automatic laboratory mill MCKA (Bühler AG, Azwil, Switzerland, diameter of grid 118–180 μm) to achieve GF flour. In particular, GF wheat kernels were decorticated by removing the external part of the fiber and grinding the endosperm.

Control flour (without technological treatments, CF) and Gluten Friendly flour (GFF) were used for bread production.

### 2.2. Chemical Composition of Flours

Protein and moisture content, as well as water absorption capacity, were determined using a Perten Inframatic analyzer (Model 9140, SE-126 53, Hägersten, Sweden) and employing the official American Association of Cereal Chemists (AACC) methods [31]. Dry gluten content and gluten index were also measured using the Glutomatic 2200 system, following the standard method 38-12.02 [31]. Alpha-amylase activity was evaluated by means of Hagberg falling numbers [31] making use of Perten model n° 1500, (Waltham, MA, USA). All analyses were performed in triplicate.

### 2.3. Determination of the Rheological and Pasting Properties

Farinographic properties were determined using a Brabender farinograph according to the AACC method 54-21 [31]. The main farinograph indexes such as water absorption percentage, dough development time (DDT), dough stability time (DST), mixing tolerance index (MTI), and elasticity were measured.

For the evaluation of the proofing performances, the extension (BU), maximum resistance to extension (BU) and extensibility (E) were measured using a Brabender extensograph with the AACC 54-10 method [31]. These parameters were evaluated after 45, 90 and 135 min of resting time.

The main alveographic parameters, tenacity (P, mm), extensibility (L), deformation energy (W) and curve configuration ratio (P/L ratio), were determined using a Chopin alveograph (NG, Villeneuve-La-Garenne, France) according to AACC International approved method 54-30.02 [31].

Finally, the main pasting properties of the flours were studied using a Brabender Amylograph (D-47055, Brabender Ohgduisburg, Duisburg, Germany) according to method 22–12 [31].

### 2.4. Bread Making

Bread was produced using the straight dough process, based on the following formulation: 100 g of flour, 2.5% yeast, 2% salt, 5% sugar and variable absorption. Baking trials were carried out under laboratory conditions to optimize baking conditions. All the ingredients were mixed in a Kenwood mixer (Model A 907 D, Kenwood Ltd., Havant, England) at 85 rpm for 2 min until a proper dough formation was obtained. Final dough temperature was 30 °C. Dough was fermented at 30 °C and 75% relative humidity for 30 min; then it was remixed, rounded and again fermented for 25 min and baked. The baking process was performed at 220 °C for 25 min using a professional electronic oven (Miwe Condo FP) until the golden brown color appeared. The resulting bread samples were allowed to cool to room temperature (25 °C) for 2 h before analyses.

### 2.5. Bread Crumb Cell Analysis

Two-dimensional cross-sectional images of the crumb structure were acquired using a flatbed scanner (HP Scanjet 4400 c) with a resolution of 300 dpi and saved in the TIFF file format. The images were analyzed using ImageJ software according to Gonzales-Barron and Butler [32]. The total number of cells, average cell/mm^2^, average diameter per mm^2^ and circularity were measured. The characterization of the crumb structure was performed in triplicate.

### 2.6. Sensory Evaluation

Sensory analyses were performed to gain information on the acceptability of several features such as the overall appearance, texture, crust color, crumb color, mouth feel, crumb stability, taste, aroma and overall acceptability. The panel used a 9-point hedonic scale where 1 = dislike extremely to 9 = like extremely, as also used in similar panels for bread quality evaluation [33,34]. A total of 30 panelists were recruited among bread makers, having many years of experience in creating, developing and tasting different kinds of bread. By recruiting bread-makers, rather than a panel consisting of habitual consumers of bread or untrained people, as is commonly employed in other experiments [33,35,36,37], allowed us to significantly reduce the time needed for training specifically focused on bread texture, taste, aroma, appearance and overall acceptability. However, prior to the assessment the panel was trained for descriptive sensory and analysis testing for a total of 20 h using different bread types. All the panelists exhibited a high capability to discriminate different intensities of the aforementioned sensory characteristics and to differentiate preliminary samples of bread. Each panelist separately performed the evaluation of three different samples: Gluten Friendly bread, control bread and a commercial gluten-friendly bread (CGFB).

### 2.7. Statistical Analysis

All determinations were performed in triplicate. Analysis of variance (ANOVA) and Tukey’s test (significance level *p* ≤ 0.05) were used to determine the significance of the data. All statistical analyses and graphical representations were performed in STATISTICA 7.0^®^.

## 3. Results and Discussion

### 3.1. Chemical Composition of the Flours

Gluten Friendly Flours (GFF) were analyzed chemically and the results are given in Table 1. The wheat flour moisture analysis results ranged from 12.1 for CF to 13.2 for GFF. The dry gluten content was decreased in GFF compared to CF. The decrease was about 90% and was statistically significant (*p* < 0.05). Furthermore, results showed that the isolation of gluten from GFF was not possible and the gluten index for GFF samples was 0. Alpha amylase activity (falling number), ranged from 345 for GFF to 350 for control flours (Table 1) and did not differ significantly (*p* < 0.05), indicating very low amylolytic activity in both samples. Dry gluten content and gluten index results suggest that “Gluten Friendly™” technology induces a significant modification of gluten proteins and this could be in accordance with different studies present in the literature that underline how the application of high temperature can denature gluten proteins, reducing their capacity to form gluten. Nevertheless, Lamacchia et al. [25] and Landriscina et al. [26] have already shown that the “Gluten Friendly™” technology involves a wise combination of temperatures with other important parameters (moisturizing, evaporation, resting, etc.), which could cause a change in the gluten protein configuration that allows them to become soluble in aqueous saline solution. In particular, Lamacchia et al. [25] and Landriscina et al. [26] showed that the “Gluten Friendly™” technology causes a rearrangement of the secondary and tertiary structure of the gluten proteins, involving a different spatial conformation of the sequences, including the so-called antigenic ones. These changes, which are not visible by SDS-PAGE [25] with and without reduction, may allow a new kind of aggregation among different classes of wheat endosperm proteins, through hydrophobic and/or ionic interactions that are only visible in immunofluorescent microscopy [26]. Furthermore, the same works show that the rearrangement of some of the gluten protein structure may involve the exposure of charges. Therefore, the rearrangement of the secondary and tertiary structure and the exposure of charges may determine the solubility of proteins in GFF, increasing the electrostatic repulsion and breaking the hydrogen bonds. On this basis, GF gluten cannot be isolated by the classical gluten index method. Furthermore, Table 1 shows that there was not any difference between GFF and CF in terms of alpha amylase activity. This confirms that the complex technological strategy involved in the “Gluten Friendly™” process, such as the contribution of heating, resting, hydration, time, etc., is not capable of causing protein denaturation in flours. On the contrary, if GF technology had caused protein denaturation (due to heating or other involved factors) a significant difference in alpha amylase activity—a very heat-sensitive enzyme—would have been detected between GFF and CF.

### 3.2. Rheological Properties of the Dough

With the aim of assessing the technological performances of the flours, rheological tests were applied to GFF and CF. First, the determinations of the mixing performance and of the farinographic properties are shown in Table 2.

GFF samples absorbed more water than CF samples (63.1% to 57.2%, respectively) to form doughs showing 500 BU. As explained by Lamacchia et al. [25] and Landriscina et al. [26], the application of GF technology could induce the rearrangement of the structure of some gluten proteins, leading to new exposure of charges and the breaking of the hydrogen bonds among glutamine proteins, which in turn become available to form new hydrogen bonds with water. This promotes the need for a higher percentage of water in GFF for the complete development of a cohesive and viscoelastic dough with optimum gluten strength. Qu et al. [38], who performed a microwave treatment on wheat flours for more than 20 s, were unable to measure any farinographic index, which may lead to the hypothesis of a complete gluten degradation. These first results are in agreement with other farinograph indexes. Specifically, dough development time (DDT) and dough stability time (DST) significantly (*p* < 0.05) decreased in GFF (1.6 and 2.1 min, respectively) in comparison with CF (8.6 min and 5.2 min). Contrarily, the mixing tolerance index (MTI) was statistically higher (*p* < 0.05) in GFF than in CF, with values of 97.4 ± 0.6 BU and 35.2 ± 0.5 BU. It is widely recognized that a strong gluten network takes longer to develop before reaching 500 BU, and it is stable for a longer time. In the case of GFF, the dough development was rapid, whereas the protein network stability was weaker, causing the reduced values of DDT and DST. In addition, the proofing performances, based on measurements of the main alveograph and extensograph properties of GFF and CF, are reported in Table 3. Treated flours (GFF) showed higher values of P (tenacity) and P/L (tenacity/elasticity) than the control flours. On the other hand, control flours revealed significant higher values of L and W (dough strength) than GFF flours. Specifically, CF showed values of 175.8 ± 0.7 J × 10^−4^ and 0.63 ± 1.5, respectively for W and P/L alveographic indices. Considering the standards adopted by the Italian market, CF may be classified as ‘ordinary bread-making wheat’, whereas GFF is not included in any ordinary category, mainly due to its very high P/L value. To better understand and explain this behavior, the results of extensograph tests are also shown in Table 3.

The results indicated remarkable differences between CF and GFF samples. The area of the extensograms, the resistance to extension and the maximum resistance to extension were significantly higher in GFF with respect to CF, whereas the extensibility decreased by more than 50% in GFF than CF, confirming the reduced values highlighted during alveographic testing. Similar results were found by Li et al. [39], who also found a negative effect of microwave treatment duration on dough extensibility. What is more, when resting time increased from 45 min to 135 min, the resistance to extension increased for GFF, and the extensibility decreased from 81.2 ± 1.3 mm for 45 min to 77.2 ± 1.5 mm for 135 min (Table 3), attesting that an increase of resting time could enhance the resistance of the treated dough to deformation, while reducing its extensibility. Preteston and Hoseney [40] classified flour samples based on the area of extensograms with values less than 80 cm^2^, which indicate weak flours; those with areas of 80–120 cm^2^, which can be classified as medium; those with areas of 120–200 cm^2^, which can be classified as strong; and those with areas above 200 cm^2^, which can be classified as very strong. Accordingly, the obtained area of extensograms allowed us to classify the Gluten Friendly Flour and the Control flour as weak.

The significant decrease (*p* < 0.05) in the elasticity of the treated flour samples is consistent with the observation that breaking the hydrogen bonds between gluten proteins and conformational changes induced by microwave treatment in the kernel allow polymers in the flour to be hydrated but not to form a high amount of well-known “loop and train” structures, responsible for dough elasticity and typical of high molecular weight (HMW)-glutenin polymers, as extensively reported by Lamaccchia et al. [17].

### 3.3. Pasting Properties of the Dough

The amylograph test was performed with the aim of obtaining more information relevant to the final step in bread making, when the transition from the foam state to the sponge state of the leavened dough occurs [41]. Amylograph results of wheat flour samples are shown in Table 4.

The pasting temperature at the initial stage of gelatinization ranged from 61.6 °C to 60.2 °C for CF and GFF, respectively, whereas at the end of gelatinization process temperatures were 91.3 °C and 89.5 °C. In addition, the maximum viscosity assessed during the experiments ranged from 995.4 to 1010.5 AU for CF and GFF samples, respectively. However, statistical analyses showed no differences between the flours, suggesting that the technological behavior of starch was not affected by microwave treatments. Pasting properties of flours treated with microwaves have been investigated by other authors, who reported contrasting results. Li et al. [39] noted no significant differences in the pasting temperature of treated flour, in agreement with our findings, although peak viscosity increased substantially, indicating an increased swelling power of the starch granule. However, Stevenson et al. [42] reported a decrease in the paste viscosity of microwaved corn starch, probably caused by the impact of non-starch components. In addition, other studies revealed a different behavior of wheat starch after microwave (MW) treatments, which influenced the structure and arrangement of starch molecules, leading to an increase in pasting temperatures and peak viscosities [38,43]. However, our results are in accordance with previous research, indicating that starch granules did not differ in number and shape in Gluten Friendly™ grain and control grain samples, and they were homogeneously dispersed in the continuous protein matrix [26].

### 3.4. Bread Crumb Image Analysis

**Figure 1:**
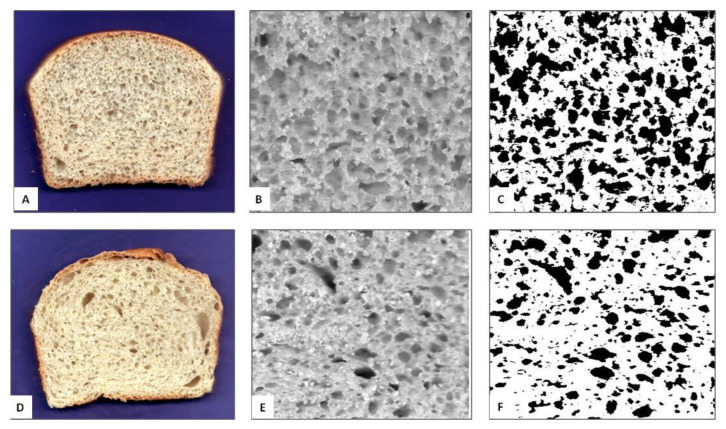
Representative images of the bread samples, regions of interests and the binarized images employed for morphological characterization of crumb structure. (**A**,**D**) represent CB and GFB, respectively; (**B**–**E**) and (**C**–**F**) represent the regions of interest (ROIs) and binarized images for CB and GFB, respectively.

In Figure 1 representative images of the bread samples, the regions of interest (ROIs) and the binarized images employed to compute the most important morphological indexes of the crumb structures are reported. Additionally, in Table 5 the results of the image analysis of bread crumb structure are shown.

Overall, the obtained data show only minor differences between the samples. More specifically, the numbers of cells, mean cell area and circularity did not show any statistical differences, although the value of cell density was higher (2259.6 ± 1.8 cells/mm^2^) for GFB than for CB (1856.6 ± 1.1 cells/mm^2^). As previously attested by similar results concerning the pasting properties of flours, which demonstrated a good attitude of both CF and GFF for bread making process, the overall results relating to bread crumb structure confirm the ability of GFF to form a leavened dough and to retain its structure during baking.

### 3.5. Sensory Evaluation of Bread Samples

The results of the sensory evaluation performed on Gluten Friendly™ bread, control breads and a commercial gluten free bread are shown in Table 6. Apart for crumb color, the samples prepared using Gluten Friendly™ flour did not show significant differences from the controls for all investigated sensorial attributes.

Another important parameter for consumers’ acceptance is the mouth feel, a property that is tightly interrelated with the morphological properties of crumb cells, the solid phase of bread samples [44]. The evaluation of the mouth feel of bread samples showed no statistical difference between GFB and CB. In general, the panel was not capable of discriminating and differentiating between GFB and CB for the majority of sensory characteristics such as for appearance, color, mouth feel, crumb stability, texture, taste, aroma and overall acceptability. Contrarily, it is worth noting the higher score of GFB when compared with a commercial gluten-free bread, proving the capability of Gluten Friendly™ technology to positively tackle the challenge of obtaining breads with an excellent sensory quality but with a reduced epitope antigenicity. In fact, although several advances in the manufacturing of gluten-free products have been obtained in the last 10 years [45], the majority of the commercial products exhibit low nutritional quality and poor mouth feel or flavor [46].

## 4. Conclusions

The Gluten Friendly™ process is an innovative technology that is capable of eradicating the antigenic capacity of gluten. We have investigated and characterized the rheological and pasting properties of the flours that identify the bread making performance of wheat grains submitted to Gluten Friendly™ technology, as well as the sensory properties of the obtained bread.

GFF showed a significant reduction in the amount of dry gluten and gluten index when compared with untreated flours. This is the result of a new spatial rearrangement of gluten proteins, responsible for the increase in the exposure of charges that generate a greater solubility of protein in GFF and higher water absorption during mixing. Furthermore, GFF manifested significantly different rheological behavior, such as farinograph water absorption, alveograph P/L ratio and also extensograph energy and resistance to extension. For instance, a very high tenacity and low extensibility were observed, meaning it was not possible to classify GFF in any ordinary category when considering the common quality standards adopted by the Italian markets. However, the pasting properties did not show any differences when the kernel was applied to Gluten Friendly™ technology. In addition, when considering the microstructure of bread samples, the main morphological parameters of the pores were not statistically different for both GFB and CB. Finally, and despite the above changes in the rheological properties of the gluten, the sensory evaluation demonstrated highly appreciated products. The panelists were unable to discriminate between GFB and CB, evaluating GFB with a high score when compared with a commercial gluten free product. This result proves the potential for the use of GFF for the production of high-quality bread.

## Figures and Tables

**Table 1:** Chemical composition of control flour (CF) and Gluten Friendly™ flour (GFF) ^1^.

Samples	Moisture (g/100 g)	Protein (g/100 g)	Dry Gluten (g/100 g)	Gluten Index	Alpha Amylase Activity (Falling No)
**CF**	12.1 ^a^	12.03 ^a^	7.5 ^a^	80	345 ^a^
**GFF**	13.2 ^b^	12.01 ^a^	1 ^b^	NA ^2^	350 ^a^

^1^ Mean values in the same column followed by a different letter are significantly different (*p* ≤ 0.05). ^2^ NA: not applicable.

**Table 2:** Farinographic characterization of control flour (CF) and Gluten Friendly™ flour (GFF) ^1^.

Samples	Water Absorption (%)	Dough Development Time, DDT (min)	Dough Stability Time, DST (min)	Mixing Tolerance Index, MTI, (BU)
**CF**	57.2 ^a^ ± 1.3	5.2 ^a^ ± 0.2	8.6 ^a^ ± 0.4	35.2 ^a^ ± 0.5
**GFF**	63.1 ^b^ ± 1.5	1.6 ^b^ ± 0.6	2.1 ^b^ ± 0.5	97.4 ^b^ ± 0.6

^1^ Mean values ± standard deviation in the same column followed by a different letter are significantly different (*p* ≤ 0.05).

**Table 3:** Alveograph and extensograph evaluation of control flour (CF) and Gluten Friendly™ flour (GFF) ^1^.

			CF	GFF
Alveographic indices	P (mm)		52.0 ^a^ ± 1.2	164.6 ^b^ ± 1.7
	L (mm)		81.5 ^a^ ± 0.3	25.4 ^b^ ± 0.4
	W (J 10^−4^)		175.8 ^a^ ± 0.7	160.3 ^b^ ± 1.3
	P/L		0.63 ^a^ ± 1.5	6.31 ^b^ ± 1.7
Extensographic indices	Area of Extensograms (cm^2^)	45 min	45.3 ^a^ ± 1.2	45.1 ^a^ ± 1.5
		90 min	45.2 ^a^± 1.4	51.4 ^b^± 1.2
		135 min	46.4 ^a^ ± 1.1	62.5 ^b^ ± 0.8
	Resistance to extension (BU)	45 min	120.1 ^a^ ± 0.5	420.3 ^b^ ± 0.9
		90 min	126.3 ^a^ ± 0.7	520.2 ^b^ ± 0.3
		135 min	135.1 ^a^ ± 0.9	615.5 ^b^ ± 1.2
	Extensibility (min)	45 min	196.1 ^a^ ± 0.4	81.2 ^b^ ± 1.3
		90 min	191.4 ^a^ ± 2.1	79.1 ^b^ ± 1.6
		135 min	189.3 ^a^ ± 1.4	77.2 ^b^ ± 1.5
	Maximum resistance (BU)	45 min	148.1 ^a^ ± 0.9	434.6 ^b^ ± 0.5
		90 min	153.4 ^a^ ± 1.2	547.3 ^b^ ± 1.5
		135 min	161.2 ^a^ ± 0.7	648.5 ^b^ ± 0.3

^1^ Mean values ± standard deviations in the same line followed by a different letter are significantly different (*p* ≤ 0.05).

**Table 4:** Pasting properties of control flour (CF) and Gluten Friendly™ flour (GFF) ^1^.

Samples	Initial Temperature of Gelatinization (°C)	Final Temperature of Gelatinization (°C)	Maximum Viscosity (AU)
CF	61.6 ^a^ ± 1.3	91.3 ^a^ ± 0.7	995.4 ^a^ ± 1.3
GFF	60.2 ^a^ ± 0.9	89.5 ^a^ ± 1.1	1010.5 ^a^ ± 0.8

^1^ Mean values ± standard deviation in the same column followed by a different letter are significantly different (*p* ≤ 0.05).

**Table 5:** Main morphological properties of crumb structures of Gluten Friendly™ bread (GFB) and control bread (CB) ^1^.

Bread Samples	Number of Cells	Mean Cell Area (mm^2^)	Cell Density (cells/mm^2^)	Circularity
CB	291.5 ^a^ ± 0.9	0.129 ^a^ ± 0.5	2259.6 ^a^ ± 1.8	0.736 ^a^ ± 0.8
GFB	254.4 ^a^ ± 0.7	0.137 ^a^ ± 0.6	1856.6 ^b^ ± 1.1	0.697 ^a^ ± 0.2

^1^ Mean values ± standard deviation in the same column followed by a different letter are significantly different (*p* ≤ 0.05).

**Table 6:** Sensory evaluation of bread samples. Gluten Friendly™ bread (GFB), control bread (CB) and gluten-free commercial bread (GFCB) ^1^.

Attributes	CB	GFB	GFCB
Appearance	7.51 ^a^	7.14 ^a^	5.98 ^b^
Crust color	7.38 ^a^	7.27 ^a^	4.73 ^b^
Crumb color	7.82 ^a^	6.89 ^b^	5.42 ^c^
Mouth feel	8.14 ^a^	7.78 ^a^	5.84 ^b^
Crumb stability	8.31 ^a^	7.14 ^a^	6.13 ^b^
Texture	7.92 ^a^	7.52 ^a^	2.78 ^b^
Taste	7.89 ^a^	7.95 ^a^	4.65 ^b^
Aroma	8.15 ^a^	8.36 ^a^	3.78 ^b^
Overall acceptability	7.45 ^a^	7.16 ^a^	5.39 ^b^

^1^ Mean values ± standard deviation in the same row followed by a different letter are significantly different (*p* ≤ 0.05).

## Data Availability

The data presented in this study are available on request from the corresponding author.
